# Huntingtin-Associated Protein 1 in Mouse Hypothalamus Stabilizes Glucocorticoid Receptor in Stress Response

**DOI:** 10.3389/fncel.2020.00125

**Published:** 2020-06-04

**Authors:** Xingxing Chen, Ning Xin, Yongcheng Pan, Louyin Zhu, Peng Yin, Qiong Liu, Weili Yang, Xingshun Xu, Shihua Li, Xiao-Jiang Li

**Affiliations:** ^1^Brain Science and Advanced Technology Institute, Wuhan University of Science and Technology, Wuhan, China; ^2^Guangdong-Hongkong-Macau Institute of CNS Regeneration, Ministry of Education CNS Regeneration Collaborative Joint Laboratory, Jinan University, Guangzhou, China; ^3^Department of Human Genetics, Emory University School of Medicine, Atlanta, GA, United States; ^4^Department of Neurology, The Affiliated Hospital of Xuzhou Medical University, Xuzhou, China; ^5^Department of Neurology, Xiangya Hospital, Central South University, Changsha, China; ^6^Department of Neurology, The Second Affiliated Hospital of Soochow University, Suzhou, China

**Keywords:** Huntington’s disease, glucocorticoid receptor, hypothalamus, protein stability, stress

## Abstract

Huntingtin-associated protein 1 (Hap1) was initially identified as a brain-enriched protein that binds to the Huntington’s disease protein, huntingtin. Unlike huntingtin that is ubiquitously expressed in the brain, Hap1 is enriched in the brain with the highest expression level in the hypothalamus. The selective enrichment of Hap1 in the hypothalamus suggests that Hap1 may play a specific role in hypothalamic function that can regulate metabolism and stress response. Here we report that Hap1 is colocalized and interacts with the glucocorticoid receptor (GR) in mouse hypothalamic neurons. Genetic depletion of Hap1 reduced the expression level of GR in the hypothalamus. Dexamethasone, a GR agonist, treatment or fasting of mice induced stress, resulting in increased expression of Hap1 in the hypothalamus. However, when Hap1 was absent, these treatments promoted GR reduction in the hypothalamus. In cultured cells, loss of Hap1 shortened the half-life of GR. These findings suggest that Hap1 stabilizes GR in the cytoplasm and that Hap1 dysfunction or deficiency may alter animal’s stress response.

## Introduction

Huntingtin-associated protein 1 (Hap1) was first identified as an interacting partner of huntingtin (Htt), the protein product of the Huntington’s disease (HD) gene (Li et al., 1995). Both Htt and Hap1 are found to be involved in intracellular trafficking, and abnormal interaction of mutant Htt with Hap1 affects the intracellular transport of various important molecules (Gauthier et al., 2004; Twelvetrees et al., 2010; Keryer et al., 2011; Roux et al., 2012; Mackenzie et al., 2014; Wong and Holzbaur, 2014). Unlike Htt that is ubiquitously expressed in the body and brain, Hap1 is enriched in neuronal cells (N2a), suggesting that Hap1 dysfunction may contribute to the selective neuropathology in HD. However, the expression level of Hap1 in the adult mouse brain varies in different regions with the highest level in the hypothalamus and low level in the striatum, thalamus, cerebral neocortex, and cerebellum (Li et al., 1996; Page et al., 1998; Fujinaga et al., 2004). These brain regions with low Hap1 expression appear to be targets of a variety of neurodegenerative diseases (Islam et al., 2017), leading to the idea that Hap1 is protective against neuronal degeneration (Fujinaga et al., 2004; Islam et al., 2017; Wroblewski et al., 2018). However, the function of Hap1 in the hypothalamus that expresses abundant Hap1 remains unclear.

The hypothalamus plays critical roles in regulation of feeding, body weight, and metabolism (Schwartz et al., 2000; Morton et al., 2014). Consistent with the abundant expression of Hap1 in the hypothalamus, loss of Hap1 can affect feeding activity and postnatal survival and growth (Chan et al., 2002; Li et al., 2003; Sheng et al., 2006; Xiang et al., 2014). These previous findings identified the critical role of Hap1 for early development that is closely related to hypothalamic function. In adulthood, hypothalamus also regulates the vital function of body. For example, stress regulation is one of the most important functions of the hypothalamus. However, whether Hap1 is involved in stress regulation has not been investigated.

Stress response involves the activation of glucocorticoid receptor (GR) in the hypothalamus, which is a member of the nuclear hormone receptor superfamily of ligand-activated transcription factors. When stress occurs, the hypothalamus-pituitary-adrenal axis (HPA) is activated and releases corticotrophin-releasing hormone from the paraventricular nucleus (PVN) of the hypothalamus, which eventually induces glucocorticoid (GC) release from the adrenal (De Kloet et al., 2005). As lipophilic molecules, GCs cross the cellular membrane by passive diffusion and bind to the GR in the hypothalamus, resulting in its conformational change and nuclear translocation to mediate gene transcription (Oakley and Cidlowski, 2011; Ratman et al., 2013). Thus, GR’s level and stability are important for stress response. In the current study, we found that loss of Hap1 can reduce the protein level of GR in the hypothalamus. Furthermore, Hap1 depletion also affects hypothalamic GR level in response to dexamethasone (Dex) treatment or during metabolic stress caused by fasting. Our findings suggest that Hap1 is involved in stress regulation by stabilizing the GR level in the hypothalamus during stress.

## Materials and Methods

### Antibodies and Reagents

Guinea pig antibody to Hap1 (EM77) has been described previously (Li et al., 1996, 2000). We also used the following antibodies: mouse monoclonal antibodies to GR [sc-393232; Santa Cruz Biotechnology (Santa Cruz, CA, USA)] and vinculin [MAB3574; MED millipore (Billerica, MA, USA)]. Secondary antibodies were horseradish peroxidase (HRP)-labeled donkey anti-mouse, donkey anti-guinea pig, donkey anti-mouse Alexa Fluor 488 or 594, and donkey anti-guinea pig Alexa Fluor488 or 594 from Jackson ImmunoResearch (West Grove, PA, USA). Tamoxifen (TM), Dex, and cycloheximide (CHX) were purchased from Sigma (St. Louis, MO, USA). Guide RNA (gRNA) backbone vector and Cas9 plasmid were purchased from Addgene (Cambridge, MA, USA).

### Animals

Mice were housed in the Division of Animal Resources at Emory University on a 12-h light-dark cycle with lights on at 7 AM and lights off at 7 PM. We fed animals (Lab Diet 5001, 12% fat, 28% protein, 60% carbohydrate) or fasted them for 24 h with free access to water. Germline *Hap1*-knockout (KO) mice were generated in our early study (Li et al., 2003). Conditional *Hap1*-KO mice were generated by crossing the floxed Hap1 mice with Cre/Esr1 (Cre-ER) transgenic mice [B6.Cg-Tg(CAG-Cre/Esr1)5Amc/J; The Jackson Laboratory (Bar Harbor, Maine, USA)] and would delete the Hap1 gene after intraperitoneal (i.p.) injection of TM (Lin et al., 2010; Xiang et al., 2014). We bred these mice and maintained them in the animal facility under specific pathogen-free conditions in accordance with institutional guidelines. All animal experiments were approved by the Animal Care Committees of Emory University.

### TM Induction in Mice

Tamoxifen (T5648; Sigma-Aldrich) was dissolved in 100% ethanol as stock solution (20 mg/ml) and stored at −20°C before use. On the day of induction, a calculated amount of TM was mixed with corn oil, and ethanol was removed by Vacufuge plus Eppendorf (Hamburg, Germany). To induce Hap1 depletion in inducible-*Hap1* KO mice, the homozygous floxed Hap1/Cre-ER mice at 2 to 3 months of age were i.p. injected with 1 mg TM per 10 g body weight for five consecutive days. Genotyping of these mice was performed using genomic DNA extracted from the tails; we used polymerase chain reaction to amplify the mouse Hap1 DNA fragment (from 4,929 to 5,003 nt) using forward (5′-TTTTTCTGGGGAGCATACGTC-3′) and reverse (5′-ATCCGTTATCCCAGGGTCTGA-3′) primers. Primers (forward: 5′-GCGGTC GGCAGTAAAAACTATC-3′ and reverse: 5′-TGTTTCACTATCCAGGTTACGG-3′) that amplify Cre recombinase were also used to determine the presence of Cre.

### Dex Treatment

Mice were injected i.p. with 1 mg/kg at a concentration of 1 mg/10 ml of Dex (Sigma-Aldrich, D1756) or an equal volume of vehicle (0.9% saline). We then isolated mouse brains at 6 h after the injection for Western blotting and immunohistochemical analyses.

### Double-Immunofluorescence Staining

The mice were deeply anesthetized, perfused with 4% paraformaldehyde, postfixed for additional 10 h in the same fixative, and switched to 30% sucrose at 4°C. After sinking completely, brains were sectioned at 20 μm with a cryostat at −19°C and mounted onto gelatin-coated slides. The tissues on slides were washed and blocked with a buffer containing 3% bovine serum albumin and phosphate buffer saline containing 0.2% Triton X-100 (PBST; 0.2% Triton X-100 in PBS) for 1 h at room temperature. Primary guinea pig antibody against Hap1 and mouse antibody against GR were incubated with the tissue at 4°C overnight, followed by incubation with Alexa 488- or rhodamine-conjugated secondary antibodies and DAPI nuclear dye. The brain sections were examined using a Zeiss (Oberkochen, Germany) (Axiovert 200M; Germany) microscope with a digital camera (Orca-100; Hamamatsu Photonics, Bridgewater, NJ, USA) and the Openlab software (Improvision, Lexington, MA, USA).

### Western Blotting

Dissected mouse hypothalamus was homogenized in RIPA buffer [150 mM NaCl, 0.1% sodium dodecyl sulfate (SDS), 0.5% sodium deoxycholate, 1% Nonidet P-40, 50 mM Tris, 1 mM EDTA, and protease inhibitor cocktail Pierce 78430 and 1 mM phenylmethylsulfonyl fluoride (PMSF), Sigma P-7626]. Samples were sonicated for 10 s, centrifuged at 16,000× *g* at 4°C for 20 min. Equal amounts of protein were loaded on Invitrogen (Carlsbad, CA,USA) Tris-glycine (4%–12%) gels for SDS–polyacrylamide gel electrophoresis. Proteins transferred to nitrocellulose blots were blocked in 5% nonfat dry milk Nestle (Glendale, CA,USA) in PBS for 30 min and then incubated with primary antibodies in 3% bovine serum albumin/PBS overnight at 4°C. Following incubation, the nitrocellulose blots were washed, and secondary HRP-conjugated antibodies (Jackson ImmunoResearch) were added in 5% milk for 1 h. ECL-plus GE Healthcare (Little Chalfont, Buckinghamshire, UK) and KwikQuant Imager Kindle Biosciences (Greenwich, CT, USA) were then used to reveal immunoreactive bands on the blots.

### Coimmunoprecipitation

Mouse hypothalamus tissue was lysed in NP40 buffer (50 mM Tris pH 7.4. 50 mM NaCl, 0.1% Triton X-100, 1% NP40, and protease inhibitor cocktail Pierce 78430 and 1 mM PMSF, Sigma P-7626). The lysate was centrifuged at 15,596× *g* at 4°C for 15 min. The supernatants were precleared by incubation with an excess of protein A agarose beads (Sigma-Aldrich) at 4°C for 2 h with gentle rocking. Supernatants (1 mg) were then collected and incubated with 2 μg anti-GR antibody at 4°C overnight. Next, 15 μl of protein A beads was added for an additional hour to pull down the endogenous GR. Beads were spun down and washed three times with the lysis buffer. After final wash, SDS loading buffer was added to the samples, and the immunoprecipitation products were detected by Western blotting using guinea pig anti-Hap1 antibody (EM77) and mouse anti-GR antibody.

### CRISPR/Cas9 Targeting

In order to remove Hap1 in N2a cells, we designed gRNAs using the CRISPR design tool^1^. The gRNA (5′-atggacccgctacgtattcc-3′, PAM: AGG) targeting exon 1 of *Hap1* gene was screened with the lowest off-target effect. The gRNA is expressed under the U6 promoter in an adeno-associated virus (AAV-9) vector that also expresses red fluorescent protein (AAV-Hap1-gRNA) under the CMV promoter, and Cas9 is expressed in another AAV-9 vector under the CMV promoter (AAV-CMV-Cas9). Mouse N2a cells were cotransfected with Hap1 gRNA and Cas9 plasmids or transfected only with Hap1 gRNA plasmid as a control using Lipofectamine 3000 reagent (Invitrogen). After 48 h, Western blotting was used to examine the effect of removing Hap1.

### CHX Chase Assay

N2a cells were transfected with plasmids expressing Cas9 and Hap1 gRNA. At 24 h following transfection, cells were treated with 50 μg/ml of CHX (Sigma, A6185) and collected at different time points from 0 to 36 h for Western blotting analysis.

### Statistical Analysis

Results are presented as mean ± SEM. Student *t*-test or one-way analysis of variance (ANOVA) with *post hoc* test was used to analyze the data with intragroup or intergroup. All statistical analyses were conducted using GraphPad Prism 7 (GraphPad Software, San Diego, CA, USA). A significant level was considered with *p* < 0.05.

## Results

### Coexpression of Hap1 and Glucocorticoid Receptor in the Hypothalamus

**Figure 1:**
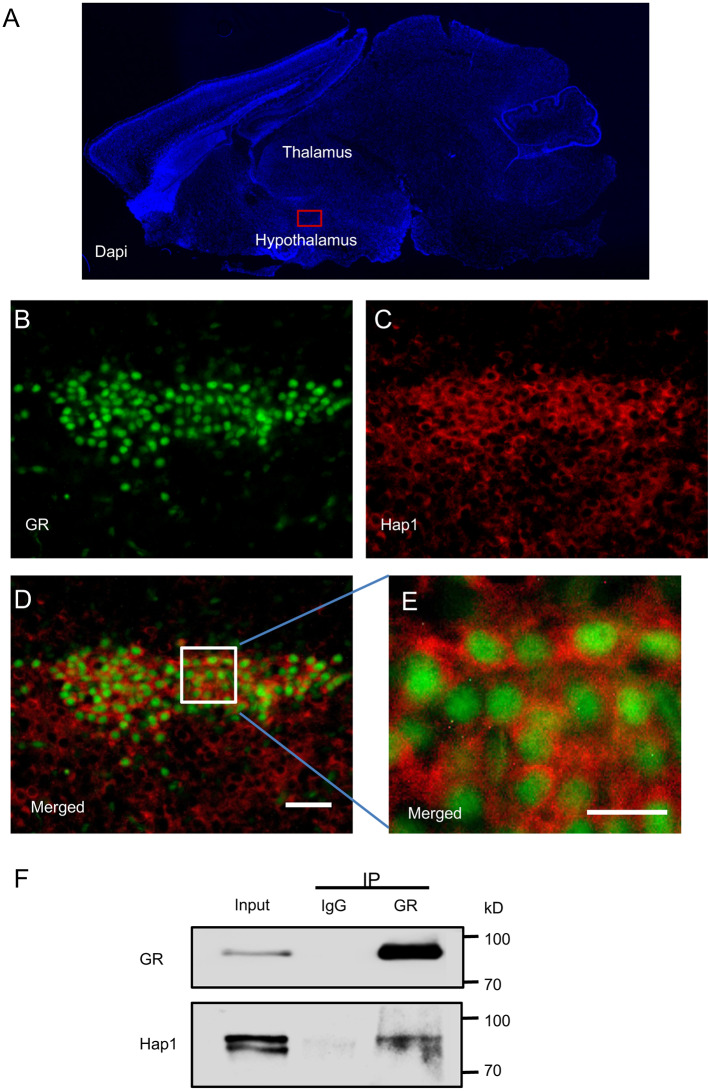
Interaction of Huntingtin-associated protein 1 (Hap1) with glucocorticoid receptor (GR). Double-immunofluorescent labeling of GR and Hap1 in the mouse paraventricular nucleus (PVN). **(A)** The sagittal section of a mouse in DAPI staining and red box indicating PVN in hypothalamus. **(B,C)** The high-power images from the red box, GR-immunoreactive cells **(B)**, and HAP1-immunoreactive cells **(C)**. **(D)** Merged image of **(B,C)**. Scale bar: 50 μm. **(E)** An enlarged image of the white square region of **(D)** showing clear double immunofluorescence for GR (green) and HAP1 (red). Scale bar: 20 μm. **(F)** Immunoprecipitation of endogenous GR from the hypothalamic tissues of WT mice showing the coprecipitation of GR and Hap1.

Hap1 is highly expressed in the hypothalamus and participates in many hypothalamic functions such as maintenance of neuronal survival and regulation of food intake and body weight (Li et al., 2000, 2003; Chan et al., 2002; Fujinaga et al., 2004; Sheng et al., 2006; Xiang et al., 2014). Stress regulation is one of the most important functions of the hypothalamus, and the stress response is mainly mediated by GR. To investigate whether Hap1 is associated with GR, an important intracellular receptor for stress response, in the hypothalamus, the distribution patterns of Hap1 and GR in hypothalamus were immunohistochemically analyzed. Double-immunofluorescent staining using anti-Hap1 (EM77) and GR antibody in sagittal mouse brain section showed that Hap1 and GR were highly expressed in the hypothalamus, especially in the PVN, which is the important neuroendocrine nucleus that regulates stress (Benarroch, 2005; Herman et al., 2016; Figures 1A–C). The GR-immunopositive cells also showed clear Hap1 immunoreactivity. However, the GR-immunoreactive product was mainly distributed in the nucleus, whereas the Hap1 staining predominantly remained in the cytoplasm (Figures 1D,E).

The predominant nuclear staining of GR could be due to the specific conformation of nuclear GR that can be easily recognized by anti-GR. Because GR is translocated from the cytoplasm to the nucleus, the colocalization of GR and Hap1 in the same hypothalamic neurons led us to examine whether both proteins are interacted with each other. By performing *in vivo* immunoprecipitation of GR from the hypothalamic tissues of WT mice and detecting Hap1 by anti-Hap1, we found that anti-GR, but not the control IgG, could coprecipitate Hap1, indicating that both GR and Hap1 are associated with each other *in vivo* (Figure 1F).

### Loss of Hap1 Reduces GR in the Hypothalamus of Postnatal Mice

**Figure 2:**
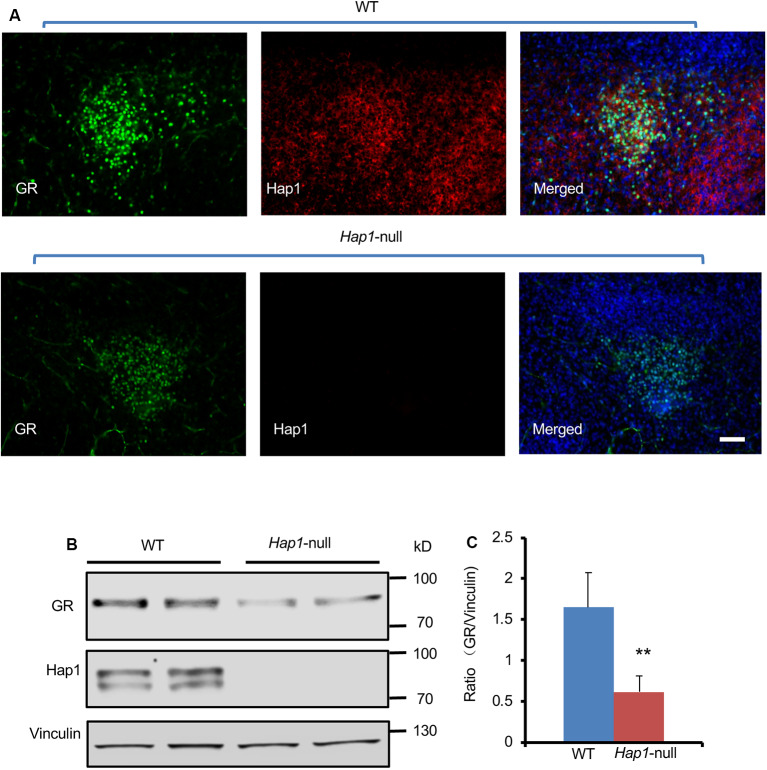
The expression level of GR in hypothalamus was reduced in Hap1 KO mice. **(A)** Representative fluorescence images of GR (green), Hap1 (red), and merged fluorescence with nuclear (blue) labeling in PVN of wild-type or Hap1-null mouse at P1. Scale bar: 50 μm. **(B)** Western blotting result showing that the GR protein level of hypothalamic tissues decreased in the knockout (KO) mice at P1 as compared to wild-type mouse. Vinculin served as an internal control. **(C)** The ratios of GR to vinculin on the Western blots were obtained from three independent experiments. Mean ± SEM; **P < 0.01; one-way ANOVA with Tukey *post hoc* test.

Given that Hap1 interacts with GR, we wanted to examine whether loss of Hap1 can affect the level of GR because Hap1 has been found to regulate the stability of intracellular receptors (Kittler et al., 2004; Twelvetrees et al., 2010; Yang et al., 2011; Xiang et al., 2014). Our previous studies have generated Hap1-null mice that are unable to survive after postnatal day 3 (P3; Li et al., 2003). Thus, we used hypothalamic tissues from *Hap1*-null mouse pups and wild-type littermates at P1 to perform immunofluorescence and Western blotting analysis. We found that lack of Hap1 reduced the GR level in the *Hap1*-KO hypothalamus (Figures 2A–C).

**Figure 3:**
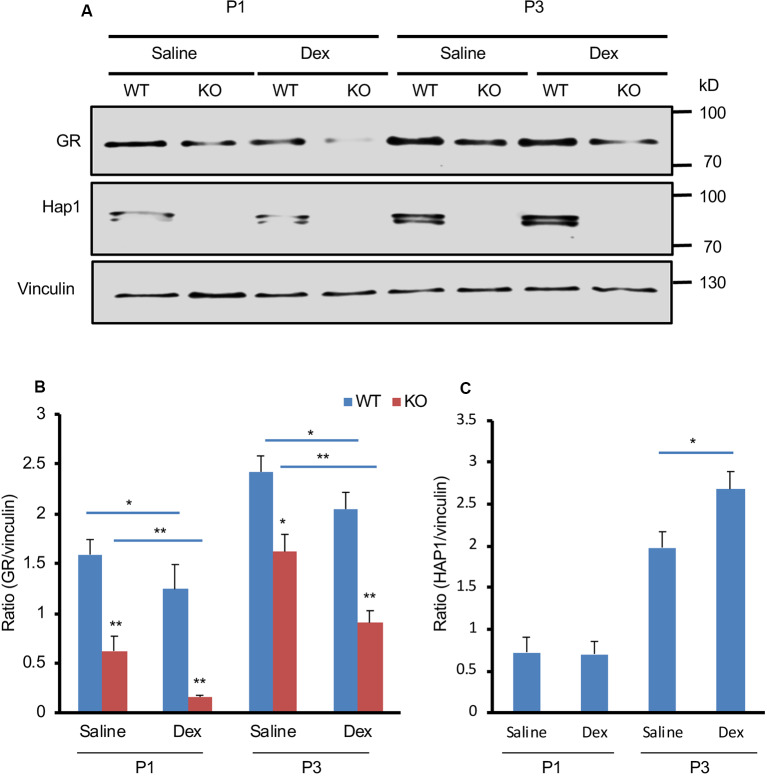
Depletion of Hap1 expression affects Dex-mediated GR expression in early development. **(A)** Western blot result showing that the GR protein level of hypothalamic tissues decreased in the *Hap1*-null mice at P1 and P3 as compared to wild-type mouse. Note that the decrease was much more dramatic 6 h after i.p. Dex administration. Vinculin served as an internal control. **(B)** The ratios of GR to vinculin were presented. **(C)** Relative levels of Hap1 (ratio to vinculin) on the Western blots were obtained from three independent experiments. Mean ± SEM; *P < 0.05; **P < 0.01; one-way ANOVA with Tukey *post hoc* test.

Administration of the GR ligand, Dex, can induce metabolic stress in newborn mice (Tuor et al., 1997; Gordon et al., 2001; Baud et al., 2005; Heine and Rowitch, 2009). To investigate the effect of Hap1 deficiency on GR under stressful condition in early development, we used i.p. injection of Dex to simulate GCs at P1 and P3 in WT and *Hap1*-KO mice. We found that GR expression is decreased in the hypothalamus in both WT and *Hap1*-KO mice 6 h after Dex injection as compared with the saline injection control. However, in the Dex-treated *Hap1*-KO mice, there was a more dramatic decrease of GR levels compared with the age-matched WT mice, especially in *Hap1*-KO mice at P1 (Figures 3A,B). Meanwhile, Hap1 level in WT mice appeared to increase after Dex administration at P3 (Figures 3A,C). The reduced GR level in the Hap1 KO hypothalamus suggests that the deletion of Hap1 can affect GR-mediated stress response in the hypothalamus at the early development stage.

### The Effect of Hap1 Deficiency on the GR-Mediated Stress in Adult Mice

**Figure 4:**
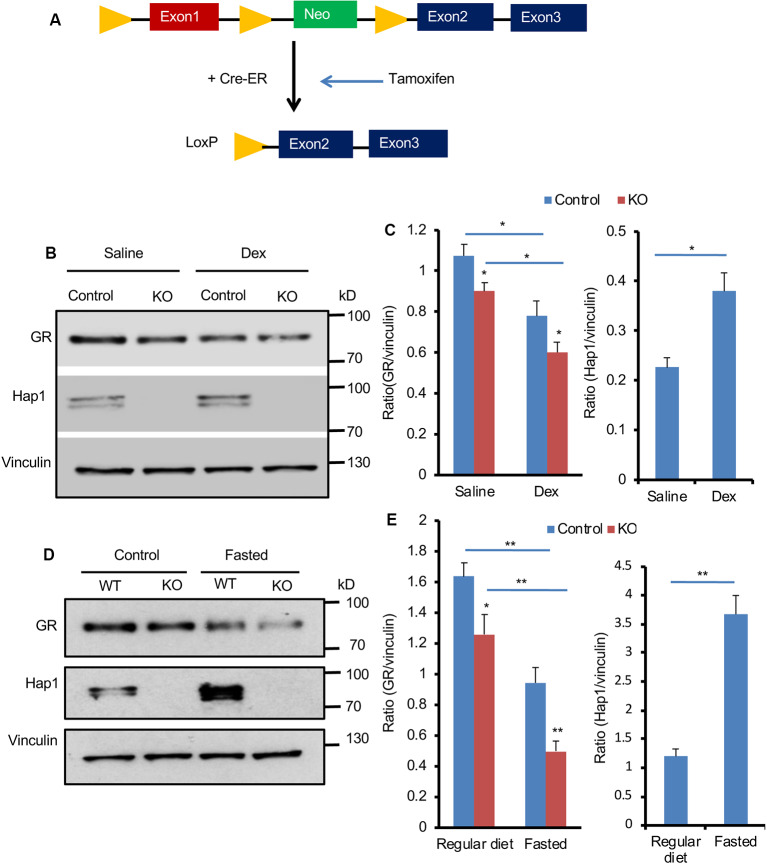
Hap1 deficiency affects the expression of hypothalamic GR in adult mice after Dex administration and fasting. **(A)** A schematic of the Tamoxifen (TM)-inducible Cre/loxP system to delete *Hap1* in adult mouse. Exon 1 of the mouse *Hap1* gene was flanked by two loxP sites and the neomycin-resistant (neo) gene for generating floxed Hap1 mice. The floxed Hap1 mice were crossed with transgenic mice expressing Cre-ER, resulting in disruption of the *Hap1* gene. **(B,C)** Representative Western blotting **(B)** and quantification **(C)** of GR and HAP1 protein levels after Dex administration, showing that the GR protein level was decreased in the hypothalamus of adult *Hap1* KO mice. The inducible *Hap1*-KO mice were treated with TM at 2 months of age. At 4 months of age, they were i.p. injected with Dex (1 mg/kg) and analyzed 6 h later. Data are presented as mean ± SEM; *P < 0.05; **P < 0.01. **(D,E)** Representative Western blotting **(D)** and quantification **(E)** of GR and Hap1 levels in the hypothalamus of control and adult *Hap1* KO mice after a 24-h fast, showing that the GR expression was further decreased in the hypothalamus of adult *Hap1* KO mice compared with WT control mice. Results were obtained from three independent experiments. Mean ± SEM; *P < 0.05; **P < 0.01.

Next, we wanted to examine whether Hap1 deficiency also affects GR in adult mouse hypothalamus. We generated conditional *Hap1*-KO mice in which exon 1 of the Hap1 mouse gene is flanked by two loxP sites that can be deleted by Cre recombination. The floxed Hap1 mice were crossed to transgenic mice that express Cre-ER ubiquitously. The crossed offspring carrying the floxed Hap1 and Cre-ER were i.p. injected with TM, which binds the cytoplasmic Cre-ER to translocate Cre to the nucleus for removing the floxed exon 1 of the Hap1 gene, leading to the disruption of the Hap1 gene at adult ages (Figure 4A). As a result, injection of TM into the floxed Hap1/Cre-ER mice at 2 to 3 months of age (adult KO) eliminated Hap1 in hypothalamus (Figure 4B).

We then used two different methods to stress HAP1 conditional KO mice. One was i.p. injection of Dex (1 mg/kg), and the other was to fast mice for 24 h. These treatments simulated different stress conditions in adult *Hap1*-KO mice. The control was floxed Hap1 mice without Cre that had also been injected with TM. Western blotting was used to detect the effect of Hap1 deletion on GR expression in the hypothalamic tissue. Injection of Dex appeared to cause further reduction of GR (Figure 4B). Quantitative analysis of the relative level of GR (ratio of GR to the loading control vinculin) on Western blots verified a greater decrease of GR in the hypothalamus of the Hap1 KO hypothalamus after Dex treatment (Figure 4C). Fasting also caused a pronounced reduction of GR in the hypothalamus when Hap1 was absent, which is evident in Western blotting results (Figures 4D,E). We noticed that Dex administration and fasting caused an increase in Hap1 expression, which was not accompanied by increased GR, in the hypothalamus of control mice (Figures 4B–E). It is possible that the cytoplasmic GR, once bound to ligand under stress, can dissociate from Hap1 and becomes less stable for degradation. Taken together, loss of Hap1 can reduce GR level under the normal physiological and stress conditions.

### Loss of Hap1 Shortens the Half-Life of GR in Cultured N2a Cells

**Figure 5:**
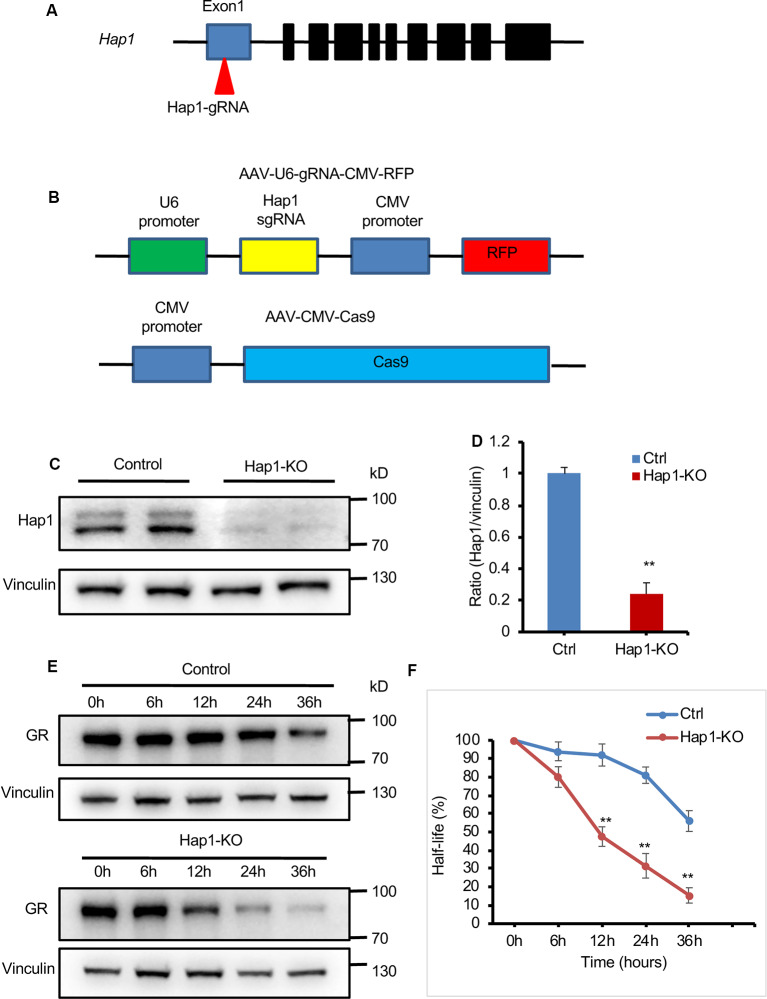
Loss of Hap1 shortened the half-life of GR in N2a cells. **(A)** Schematics of the designed Hap1-gRNA. **(B)** Schematics of AAV-Hap1-gRNA and AAV-CMV-Cas9 vectors. **(C)** Western blotting assay shows Hap1 reduction in N2a cells that were cotransfected with Hap1 gRNA and Cas9 plasmids. **(D)** Quantitative analysis of Western blotting results in **(C)**. Data are presented as mean ± SEM; **P < 0.01. **(E)** Western blot analysis of half-life of GR in transfected N2a cells that were treated with CHX for different times (0, 6, 12, 24, 36 h) to inhibit protein synthesis. **(F)** The relative protein levels of GR were assessed by densitometric analysis of their bands on Western blots, and the value at 0 h was considered 100%. Results were obtained from three independent experiments. Mean ± SEM; **P < 0.01.

The interaction of both Hap1 and GR and regulatory effect of Hap1 on GR’s level raise a possibility that Hap1 stabilizes GR via its interaction such that loss of Hap1 promotes its degradation and reduces its level. To explore this possibility, we examined the effect of Hap1 on the half-life of GR in cultured N2a. We used CRISPR/Cas9 to target the Hap1 gene (Figure 5A) via transfection of plasmids expressing Cas9 and Hap1 gRNA in order to suppress the endogenous expression of Hap1 in N2a cells (Figure 5B). To minimize potential off-targets, we used the CRISPR design tool (crispr.mit.edu) to design the gRNAs specific for the Hap1 gene. Western blotting validated the efficient reduction of Hap1 at the protein level in N2a cells, but not the control protein vinculin, supporting the specific reduction of Hap1 by Cas9/Hap1 gRNA (Figures 5C,D). We then measured the half-life of GR in the transfected N2a cells that were treated with 50 μg/mL of CHX, an inhibitor of protein synthesis. The cell lysates were collected at different time points for Western blotting analysis. The results clearly showed that GR in *Hap1*-KO N2a cells was degraded much faster than that in control cells (Figures 5E,F).

## Discussion

The hypothalamus is an important organ to regulate stress response, which involves the GR. Stress can cause GCs to be released from the adrenal, which then cross the cellular membrane to bind GR in the hypothalamus (De Kloet et al., 2005). The ligand-bound GR undergoes a conformational change and nuclear translocation to mediate gene transcription (Oakley and Cidlowski, 2011; Ratman et al., 2013). Thus, the expression level of GR in the hypothalamus is important for copying with stress. Our findings suggest that Hap1 can regulate GR at the protein level in the hypothalamus to modulate hypothalamic function for stress response.

The above suggestion is also supported by the abundant expression of Hap1 in the hypothalamus. Moreover, Hap1 expression in the hypothalamus is increased by Dex treatment and fasting, also suggesting that Hap1’s expression is inducible by stress. Our early studies have shown that elimination of Hap1 in germline can cause neuronal degeneration in the hypothalamus and postnatal death in mice (Li et al., 2003). The effect of embryonic depletion of Hap1 suggests that Hap1 is important for neuronal differentiation and survival during early development. Indeed, using conditional Hap1 KO mice that deplete Hap1 in adulthood, we confirmed that Hap1 is important for postnatal neurogenesis and that deletion of the Hap1 gene in adult mice does not induce neurodegeneration (Xiang et al., 2014). Thus, Hap1 plays distinct roles in developing and mature neurons. In the current study, we provide evidence for the additional function of Hap1, which regulates GR level in the adult mouse hypothalamus.

The regulation of GR by Hap1 appears to occur at the protein level. This is because knocking down Hap1 can promote the degradation of GR in cultured N2a. This finding fits with Hap1’s function on other intracellular receptors and molecules. For example, Hap1 has been reported to stabilize BDNF and its receptor TrkB (Yang et al., 2011; Xiang et al., 2014) and GABA(A) receptor (Kittler et al., 2004; Twelvetrees et al., 2010). Thus, regulation of GR by stabilizing it is also consistent with the function of Hap1 in stabilizing receptors and proteins during their trafficking (Rong et al., 2006; Wu and Zhou, 2009).

Adverse stress has been associated with a variety of disorders such as depression, neuropsychiatric disorders, and neurodegeneration. For example, patients with Alzheimer’s or Parkinson’s or Huntington’s disease show chronically high cortisol levels, suggesting changes occurring in controls of HPA axis (Vyas and Maatouk, 2013). Disruption of normal HPA axis activity is a major risk factor of neurodegenerative diseases and neuropsychiatric disorders in which decreased expression of GR has been documented (Oakley and Cidlowski, 1993; Karanth et al., 1997; Uys et al., 2006; Boero et al., 2018). Our findings raise a new possibility that Hap1 dysfunction in the hypothalamus may be involved in abnormal stress-associated disorders. Although further investigation is required to explore this possibility, our findings suggest that Hap1 is a promising candidate for investigating the pathogenesis of stress-related disorders that are associated with hypothalamic dysfunction.

## Data Availability Statement

The datasets generated for this study are available on request. Requests to access the datasets should be directed to rashid.giniatullin@uef.fi.

## Ethics Statement

The animal study was reviewed and approved by the Animal Care Committees of Emory University.

## Author Contributions

XC, SL, and X-JL conceived and designed the experiments. XX provided advice. XC, NX, YP, LZ, PY, QL, and WY performed the experiments and analyzed the data.

## Conflict of Interest

The authors declare that the research was conducted in the absence of any commercial or financial relationships that could be construed as a potential conflict of interest.

## Footnotes

^1^http://crispr.mit.edu
